# Dimethyl fumarate reduces TNF and *Plasmodium falciparum* induced brain endothelium activation in vitro

**DOI:** 10.1186/s12936-020-03447-7

**Published:** 2020-10-21

**Authors:** Neida K. Mita-Mendoza, Ariel Magallon-Tejada, Priyanka Parmar, Raquel Furtado, Margaret Aldrich, Alex Saidi, Terrie Taylor, Joe Smith, Karl Seydel, Johanna P. Daily

**Affiliations:** 1 Department of Microbiology & Immunology and Infectious Diseases, Albert Einstein College of Medicine, Bronx, NY USA; 2 Seattle Biomedical Research Institute, Seattle, WA USA; 3 Division of Infectious Diseases, Department of Medicine, Albert Einstein College of Medicine, Bronx, NY USA; 4 Blantyre Malaria Project, University of Malawi College of Medicine, Blantyre 3, Malawi; 5 Department of Osteopathic Medical Specialties, College of Osteopathic Medicine, Michigan State University, East Lansing, MI USA; 6 Seattle Children’s Research Institute, Seattle, WA USA; 7 Department of Global Health, University of Washington, Seattle, WA USA; 8 Present Address: Department of Research in Parasitology, Gorgas Memorial Research Institute for Health Studies, Panama City, Panama

**Keywords:** NRF2 pathway, Cerebral malaria, Human brain microvascular endothelial cell activation, IL-6, Dimethyl fumarate, Nuclear factor κb, TNF, ICAM1, EPCR

## Abstract

**Background:**

Cerebral malaria (CM) is associated with morbidity and mortality despite the use of potent anti-malarial agents. Brain endothelial cell activation and dysfunction from oxidative and inflammatory host responses and products released by *Plasmodium falciparum*-infected erythrocytes (IE), are likely the major contributors to the encephalopathy, seizures, and brain swelling that are associated with CM. The development of adjunctive therapy to reduce the pathological consequences of host response pathways could improve outcomes. A potentially protective role of the nuclear factor E2-related factor 2 (NRF2) pathway, which serves as a therapeutic target in brain microvascular diseases and central nervous system (CNS) inflammatory diseases such as multiple sclerosis was tested to protect endothelial cells in an in vitro culture system subjected to tumour necrosis factor (TNF) or infected red blood cell exposure. NRF2 is a transcription factor that mediates anti-oxidant and anti-inflammatory responses.

**Methods:**

To accurately reflect clinically relevant parasite biology a unique panel of parasite isolates derived from patients with stringently defined CM was developed. The effect of TNF and these parasite lines on primary human brain microvascular endothelial cell (HBMVEC) activation in an in vitro co-culture model was tested. HBMVEC activation was measured by cellular release of IL6 and nuclear translocation of NFκB. The transcriptional and functional effects of dimethyl fumarate (DMF), an FDA approved drug which induces the NRF2 pathway, on host and parasite induced HBMVEC activation was characterized. In addition, the effect of DMF on parasite binding to TNF stimulated HBMVEC in a semi-static binding assay was examined.

**Results:**

Transcriptional profiling demonstrates that DMF upregulates the NRF2-Mediated Oxidative Stress Response, ErbB4 Signaling Pathway, Peroxisome Proliferator-activated Receptor (PPAR) Signaling and downregulates iNOS Signaling and the Neuroinflammation Signaling Pathway on TNF activated HBMVEC. The parasite lines derived from eight paediatric CM patients demonstrated increased binding to TNF activated HBMVEC and varied in their binding and activation of HBMVEC. Overall DMF reduced both TNF and CM derived parasite activation of HBMVEC.

**Conclusions:**

These findings provide evidence that targeting the NRF2 pathway in TNF and parasite activated HBMVEC mediates multiple protective pathways and may represent a novel adjunctive therapy to improve infection outcomes in CM.

## Background

Cerebral malaria (CM), a severe complication of *Plasmodium falciparum* malaria, is associated with significant morbidity and mortality despite effective anti-malaria drug treatment with artesunate [1–7]. Paediatric survivors of CM can develop epilepsy or other neurological sequelae, including behavioural problems [4, 8–10]. To date, trials of adjunctive therapies to improve CM infection outcomes have demonstrated minimal to no improvement in morbidity and mortality [11].

CM has predominant central nervous system (CNS) clinical findings including encephalopathy, seizures and increased brain volume; this latter feature is strongly associated with death [12–15]. CNS autopsies in CM demonstrate microvascular occlusion and haemorrhages in the brain microvasculature due to parasite adherence, with accompanying neuronal degeneration [16–19]. CNS endothelial cell damage is a central pathologic feature of CM and is likely due to host and parasite induced toxicity. The CNS microvascular cytoadhesion by infected erythrocytes (IE) is mediated by the *P. falciparum* erythrocyte protein 1 (PfEMP1) family, encoded by *var* genes and expressed on the surface of IE. CM associated IE express binding domains for endothelial protein C receptor (EPCR) and intercellular adhesion molecule 1 (ICAM1) [20–24]. TNF, which is elevated during malaria infection, upregulates ICAM1 leading to higher parasite cytoadhesion levels. *P. falciparum*-IE can activate and increase endothelial cell barrier permeability [25–32]. However, most in vitro studies of the parasite contribution to pathogenesis use laboratory-adapted strains that have been maintained for prolonged periods. Limited or no work has been done with recent ex vivo clinical isolates or lab-adapted parasite lines derived from CM cases. Thus, considerable knowledge gaps remain about how CM parasite isolates activate endothelial cells to contribute to CM, and how adjunctive therapy could reduce endothelial cell activation to improve outcomes.

One potentially protective pathway in CM is the nuclear factor E2-related factor 2 (NRF2) pathway, which is a master anti-oxidant and anti-inflammatory pathway that can be therapeutically upregulated [33]. In models of cerebral vascular diseases, which share pathologic features with CM, it has been demonstrated that upregulation of the NRF2 pathway provides protection from oxidant injury and reduces infarct volume, neuronal death and cerebral edema [16, 34–38]. Dimethyl fumarate (DMF) is an FDA-approved drug which upregulates the transcription factor NRF2 to provide an anti-inflammatory and antioxidant effect in endothelial cells [39]. The effect of DMF on primary human brain microvascular endothelial cell (HBMVEC) activation was tested in an in vitro co-culture model with CM derived parasite lines and TNF treatment.

## Methods

### CM derived malaria isolates

*Plasmodium falciparum* parasite lines were derived from Malawian children with CM enrolled in a study of the clinicopathologic causes of CM run by the Blantyre Malaria Project (BMP) [1, 4]. Study subjects aged 6 months to 12 years with World Health Organization (WHO) defined CM (coma at least 1 h after termination of a seizure or correction of hypoglycaemia, asexual forms of *P. falciparum* parasites on peripheral blood smears and no other cause to explain the coma), were enrolled after informed consent was obtained from the parent or guardian. Venous blood was drawn into EDTA coated tubes. Malaria retinopathy status was determined by fundoscopic examination and brain swelling status was determined by MRI imaging, as previously described [13, 40, 41]. The patient’s clinical and demographic information was recorded. From the admission blood draw, parasite lines were obtained by in vitro limiting dilution cloning at 3 different cell concentrations (0.3 IE per well, 3 IEs per well, and 30 IEs per well). For each CM parasite line, parasites were expanded from positive wells at the lowest initial cell seeding concentration for that isolate. Following limited dilution cloning, the parasite lines were then expanded in vitro in Malawi and seed lots were frozen [42]. Cryopreserved parasites were shipped to Albert Einstein College of Medicine, NY. Institutional Review Board (IRB) approvals were obtained from the Albert Einstein College of Medicine, Michigan State University and from the University of Malawi College of Medicine Research and Ethics Committee.

### *Plasmodium falciparum* culture

Frozen stocks of CM derived parasite clones were thawed using standard methods and cultured in O^+^ RBCs at 5% hematocrit in supplemented RPMI 1640, including Albumax II (Gibco/BRL, Grand Island, NY) and cultivated under 1% O_2_, 5% CO_2_, and 94% N_2_ gas mixture and at 37 °C [43]. The parasite lines were thawed and expanded for 2–5 cycles prior to freezing and then grown for an additional 10–20 cycles until the co-culture experiments with HBMVEC. *P. falciparum* laboratory lines and CM isolates were regularly tested and confirmed free of *Mycoplasma *spp. by the MycoScope PCR detection kit (Genlantis, San Diego, CA). Two long-term cultivated parasite lines with known adhesion traits were employed: ITgICAM1 (DC17 expressing line that binds ICAM1) and IT4var19 (DC8 expressing line that binds EPCR) [22]. These parasites were cultured as above, using RPMI 1640 medium supplemented with 10% Human AB serum (Thermo Fisher Scientific, Pittsburg, PA). The parasite-induced knob-like protrusions were maintained by periodic gelatin flotation, and parasite lines were synchronized by 5% d-sorbitol lysis as needed [44].

### HBMVEC cell culture

Cerebral cortex derived HBMVECs (passage 3) were purchased from Cell Systems (Kirkland, WA) and grown in a standard 75-cm^2^ culture flask (Corning Inc, Corning, NY) coated with 0.2% gelatin (Sigma Aldrich, St. Louis, MO). The cells were maintained at 37 °C in 5% CO_2_ and grown in M199 medium (Gibco/BRL, Grand Island, NY) supplemented with 1.6 mM l-glutamine, 50 units/ml penicillin, 50 μg/ml streptomycin, 50 μg/ml ascorbic acid, 25 μg/ml heparin, 5 μg/ml bovine brain extract (Lonza, Wayne, PA), 20% calf serum, 5% human AB serum, and 7.5 μg/ml Sigma growing factor (Sigma Aldrich, St. Louis, MO). Cells were generally split when they reached approximately 90% confluency by lifting with a Trypsin/EDTA solution. HBMVEC cultures were regularly tested and confirmed free of *Mycoplasma* spp. by the MycoScope PCR detection kit (Genlantis, San Diego, CA). Cells were maintained under 5% CO_2_ at 37 °C for all experiments. HBMVEC were used between passages 7–8 for all experiments.

### Microarray processing and data analysis

For HBMVEC transcriptional analysis, cells were grown to confluence in collagen coated 6 well plates. On the day of experiment, cells were rinsed with fresh media and incubated with TNF (10 ng/ml), (PEPROTECH Inc., Rocky Hill, NJ), media alone or vehicle control (0.1% DMSO, ATCC, Gaithersburg, MD) for 6 h. To examine the effect of DMF, a set of cells were preincubated with DMF (50 μM) for 1 h prior to the addition of TNF stimulation for 6 h. HBMVEC supernatants were collected and stored for cytokine measurement, cells were rinsed with cold PBS and lysed/homogenized in Trizol (Thermo Fisher Scientific, Pittsburg, PA) for RNA extraction. RNA was purified using the Midi Total RNA Purification kit (Thermo Fisher Scientific, Pittsburg, PA), and DNase I treated. RNA yields were quantified using a Nanodrop Spectrophotometer and quality assessed with the Bioanalyzer RNA 6000 Nano assay (Agilent Technologies Inc., Wilmington, DE).

HBMVEC RNA was processed by standard protocols and hybridized to a human Clariom S microarray (Thermo Fisher Scientific, Pittsburg, PA) which contains probes for 25,000 human gene transcripts at the Genomics Core of Albert Einstein College of Medicine. Gene expression profiles were generated using a robust multi-array average algorithm followed by quantile normalization and batch correction (Transcriptome Analysis Console, Thermo Fisher). Three independent experiments, each done in triplicate, were batch corrected for day of experiment. Differential gene expression between experimental conditions was determined by calculating the p value by Student’s t test and false discovery rate (FDR). Significantly differentially expressed genes that have a FDR ≤ 0.01, and fold change > 2 are reported. p value is calculated by a Fisher’s exact test right tailed using Ingenuity Pathway Analysis (IPA) (Qiagen, Redwood City, CA) to determine if the dysregulated genes are enriched in a canonical pathway. Pathways with at least 8 genes are reported. Z-score statistics indicate the activation state of the pathway.

### DBLα tag and *var* gene expression analysis of CM parasite lines

To characterize the *var* gene expression in the lab-adapted CM parasite lines, an aliquot of 250–500 μl pellet at 4–5% ring-stage parasitemia from each parasite line was collected, one day prior to the HBMVEC cytoadherence experiments. Total RNA was extracted from samples resuspended in 3–6 ml Trizol LS (Thermo Fisher Scientific, Pittsburg, PA) and stored at − 80 °C. RNA was purified with RNeasy Micro Kit (Qiagen, Germantown, MD) following manufacturer’s instructions and contaminating genomic DNA was eliminated with DNase I treatment. cDNA was synthesized by reverse transcription using Multi-Scribe Reverse Transcriptase Kit and random hexamers (Thermo Fisher Scientific, Pittsburg, PA) at 25 °C for 10 min, 48 °C for 30 min, and 95 °C for 5 min. cDNA samples were considered DNA free when fluorescence remained at baseline after 30 cycles of quantitative PCR (qPCR) with *seryl-t-RNA synthetase* primers [45]. DBLα tags were PCR-amplified using the previously described varF_dg2 and brlong2 primers [46]. Between 40 and 50 DBLα amplicons were sequenced from each CM parasite line. To classify DBLα tags into predicted CD36 or EPCR binders, BLAST searches were conducted against a custom library of 521 annotated *var* genes, as described previously [23]. Predictions were considered high confidence when ≥ 4 of the top 5 hits were the same type. This methodology likely underestimates the true level of DC8-EPCR binders (group B/A chimeric *var* gene) as it can misclassify these DBLα tags as group B/C (CD36 binders). Additionally, the number of cysteine residues in the DBLα amplicons were quantified. Previous work has established that Group A *var* genes have 2 cysteine residues (C2 group) and group B and C *var* genes encode 3–5 cysteines with most having 4 cysteines (C4 group) [47].

### *Plasmodium falciparum* genotyping

To determine the number of *P. falciparum* genotypes in the laboratory adapted CM parasite lines DNA was isolated from IE lysed with 0.15% saponin solution and purified by phenol/chloroform organic solvents [48], and MSP1-PCR was performed as described [49]. Additionally, MSP2-PCR was performed with the M2-OF and M2-OR primers [50] and five amplicons were sequenced from each CM parasite line.

### *Plasmodium falciparum* cytoadherence to HBMVEC

HBMVEC were grown as a monolayer on Biocoat collagen I-coated 8-well chamber slides (Thermo Fisher Scientific, Springfield Township, NJ) until 90% confluence. Trophozoite stage IEs were purified by magnetic columns (Miltenyi Biotec, Gaithersburg, MD), adjusted to 3 or 10% parasitemia and 1% hematocrit in binding media (RPMI 1640, HEPES, BSA, pH 6.4), and added to HBMVEC monolayers in duplicate wells. Co-cultures were incubated for 1 h at 37 °C under semi-static conditions, with constant horizontal agitation at 100 rpm [51]. Unbound IE were gently washed off in pre-warmed binding media, cells were fixed in 2% glutaraldehyde for 2 h at room temperature and stained in 10% Giemsa for 30 min. Number of bound IE to HBMVEC were quantified in 8 fields/well in duplicate wells. IE were manually counted and HBMVEC were automatically counted using a software assisted counter (NIS-Elements Imaging Software, Nikon, Melville, NY). For cytoadherence inhibition assays of the lab lines, monolayers of HBMVEC were incubated with monoclonal antibodies to ICAM1 mAb 15.2 (10 µg/ml) or antibodies to EPCR mAb 252 (50 µg/ml) (Novus Biologicals, Centennial CO), and their corresponding IgG isotype controls, at 37 °C for 30–45 min prior binding assays. All experiments were carried out in three independent experiments.

Data is reported as IE numbers/500 HBMVEC. Laboratory strains ITgICAM1 (ICAM1 binder) and IT4var19 (EPCR binder) served as binding controls, respectively [20, 22]. For cytoadherence under inflammatory conditions, HBMVEC were stimulated with TNF (10 ng/ml) for 20 h prior to binding assays. Binding assays were conducted at 37 °C for 30–45 min. All experiments were carried out in three independent experiments.

### Endothelial cell activation assays

HBMVEC activation was measured by the quantification of interleukin-6 (IL-6) levels into cell supernatant under various experimental conditions using standard enzyme-linked immunosorbent assay (ELISA) methods [52]. Parasites were enriched for > 95% trophozoite stage by magnetic columns and added to HBMVEC confluent monolayers in a 96 well plate in M199 complete medium. To determine an optimal IE:HBMVEC stimulation ratio, 2× serial dilutions from 200:1 to 6:1 IE:HBMVEC ratios were tested. HBMVEC were cultured with uninfected erythrocytes as a negative control or TNF (10 ng/ml) (PEPROTECH Inc., Rocky Hill, NJ) as a positive control. The cells were incubated at 37 °C in 5% CO_2_ for 6 and 24 h in triplicate. Cell supernatants were collected at each time point, centrifuged at 400×*g* for 5 min at 4 °C to remove cellular debris, and stored at − 80 °C. To evaluate the effect of DMF on IE-mediated endothelial cells activation, HBMVEC were pre-treated with 50 μM of DMF for 1 h before the co-culture with IE. In all experiments where DMF effect was evaluated the IE:HBMVEC ratio used was 12:1 for the CM derived isolates and 25:1 for the IT4var19 parasite line.

To study the effect of DMF on endothelial activation, monolayers of HBMVEC were pretreated for 1 h with DMF (50 μM) or vehicle DMSO (0.1%) and then stimulated with TNF (at 1 and 10 ng/ml) in complete M199 medium. To evaluate the effect of DMF administered as co-treatment with TNF, HBMVECs were simultaneously treated with DMF (50 μM) and TNF (1 and 10 ng/ml) or the vehicle control DMSO (0.1%) and TNF for 24 h. Cell supernatants were collected, centrifuged at 400×*g* for 5 min at 4 °C to remove cellular debris. 80 μl of the clarified media was recovered and stored in single-use aliquots for cytokine measurement at − 80 °C.

### Cytokine assessment by ELISA of HBMVEC supernatants

Concentrations of IL-6 in the cell culture supernatants were measured by commercially available human IL-6 ELISA set (BD Biosciences, Palo Alto, CA). The ELISAs were performed according to manufacturer’s instructions. Flat-bottom 96-well microplates were coated with anti-human IL-6 monoclonal antibody at 4 °C overnight. Cell supernatants were assayed as neat or one- to fourfold dilutions. 3,3′,5,5′-tetramethylbenzidine (TMB) substrate reagent set (BD Biosciences) was used to detect the streptavidin–horseradish peroxidase (BD Biosciences) reaction and optical densities were measured at 450 nm with 750 nm wavelength corrections in a microplate reader. The experiments were carried out in triplicate wells in at least three independent experiments.

### Flow cytometry

Expression of endothelial cell surface receptors was determined by flow cytometry using antibodies to ICAM1 (Bio-Rad, Raleigh, NC), VCAM1, EPCR, E-selectin and CD31 (all purchased from R&D systems, Minneapolis, MN).

HBMVEC monolayers were rinsed with PBS and incubated with pre-warmed non-enzymatic cell detachment solution (Sigma, Burlington, MA) for 15–30 min at 37 °C and gently detached with a cell scraper. HBMVEC were collected in polystyrene tubes, rinsed with cold PBS at 300×*g* for 4 min at 4 °C and transferred to v-bottom 96 well plates. Cells were incubated with live/dead violet dye (Thermo Fisher Scientific, Fair Lawn, NJ) in 100 μl of FACs buffer (PBS, 10% FBS, 0.2% EDTA) for 30 min and rinsed with cold PBS. HBMVECs were incubated with human Fc receptor blocking (eBioscience, Thermo Fisher Scientific, Fair Lawn, NJ) for 20 min, and multiplexed primary antibodies and fluorochrome conjugated antibodies were added to HBMVEC and incubated for 1 h. Excess antibody was washed and HBMVEC incubated with fluorochrome conjugated secondary antibodies for 30 min. Cells were washed twice with cold PBS for 5 min, centrifuged at 300×*g* for 5 min at 4 °C and fixed with IC fixation buffer (Thermo Fisher Scientific, Fair Lawn, NJ) for 20 min. Excess fixative was washed and cells were resuspended in 1% BSA PBS for analysis. Cell cytometry data was acquired on LSRII flow cytometer and analysis performed with FlowJO software. Assays were performed in 3 to 5 independent experiments.

### Nuclear translocation of nuclear factor κB (NFκB)

Modulation of the endothelial NFκB nuclear translocation by DMF and TNF was evaluated with confocal immunofluorescence. HBMVEC were deposited onto lysine and collagen-coated glass bottom dishes (MatTek Corporation, Ashland, MA) and cultured to confluency. HBMVEC monolayers were stimulated with TNF (10 ng/ml) or left unstimulated in M199 medium alone for 30 min or pre-treated with DMF (50 μM) or vehicle DMSO (0.1%) for 1 h prior to TNF stimulation. Cells were rinsed and then fixed with 4% paraformaldehyde (PFA) (Sigma, Burlington, MA) for 30 min, permeabilized and blocked with 0.2% Triton X-100 (Sigma, Burlington, MA), 10% normal goat serum (Thermo Fisher Scientific, Fair Lawn, NJ), 1X PBS and 0.05% sodium azide for 1 h at room temperature. They were incubated overnight with anti-NFκBp65 (Santa Cruz Biotechnology, Dallas, TX) at 4 °C followed by Alexa Fluor-488 (1:300, Invitrogen Carlsbad, CA), and the F-actin probe Alexa Fluor 680 Phalloidin (1:40, Thermofisher Scientific, Fair Lawn, NJ) for 1 h at room temperature. Cells were fixed with 4% PFA and nuclei were stained with 4′-6-diamidine-2-phenylindole (DAPI) (Vector Laboratories, Burlingame, CA), and mounted with antifade reagent ProLong Gold (Molecular Probes, Thermo Fisher Scientific, Fair Lawn, NJ). Images were collected with a Leica SP2 inverted confocal microscope (Leica Microsystems) under a 63X oil-immersion objective. Experiments were carried out in at least three independent instances.

### Statistical analysis

Statistics were performed using Prism 6 (GraphPad Software). Values reported are the mean ± SEM. All analyses utilized an alpha = 0.05. One-way or two-way ANOVAs with either Sidak’s or Turkey’s post hoc comparisons were used to compare samples where appropriate, as indicated in the figure legends.

## Results

### Effect of DMF on TNF-stimulated HBMVEC transcription, IL-6 release, and NFκB localization

**Fig. 1:**
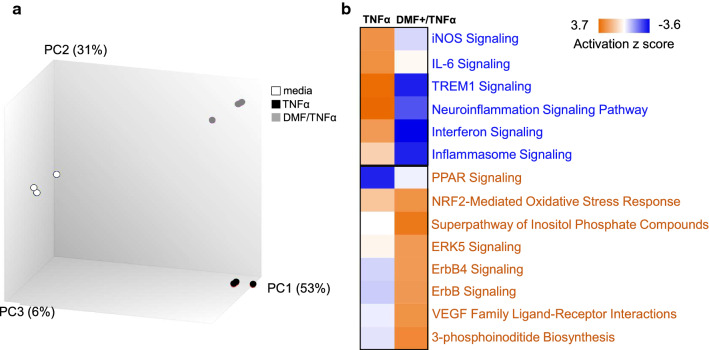
DMF pretreatment of TNF stimulated HBMVEC modulates multiple inflammatory pathways. **a** Principal component analysis (PCA) of HBMVEC endothelial cell transcriptomes treated with TNF (10 ng/ml) for 6 h; DMF pretreatment (50 µM for 1 h) prior to TNF stimulation or untreated, segregate by experimental group. **b** Heat map of pathways differentially expressed between TNF versus DMF pretreated TNF stimulated HBMVEC. p < 0.005, FDR ≤ 0.01, FC > 2. Experiments done in triplicate. *DMF* dimethyl fumarate

HBMVEC transcriptional profiling to define the global effects of DMF treatment on TNF activated cells was conducted. Gene expression profiles of HBMVEC subjected to TNF compared to DMF pre-treatment, and media alone (control) segregated by experimental group in a Principal Component Analysis (PCA) (Fig. 1a). TNF treatment of HBMVEC compared to media alone, altered the expression of 514 genes (fold change > 2, FDR ≤ 0.01, p < 0.005, see Additional file 1 for complete gene list). TNF upregulated cell adhesion molecules, including intercellular adhesion molecule 1 (ICAM1) and vascular cell adhesion molecule 1 (VCAM1). It also induced inflammatory mediators, such as chemokine (C-X-C motif) ligand 6 (CXCL6), Interleukin 1 Beta (IL1β) and NFκB2. TNF treatment upregulated the Neuroinflammation Signaling Pathway, High Mobility Group Protein 1 (HMGB1), NFκB, Triggering Receptor Expressed on Myeloid cells 1 (TREM1), Transforming Growth Factor Beta (TGFβ) and IL-6 signaling pathways, and downregulated the Peroxisome Proliferator-activated Receptor (PPAR) Signaling Pathway when compared to controls (Ingenuity Canonical Pathways, p < 0.01, Z score at least 2 SD above the mean, see Additional file 2 for complete list of pathways).

To determine the effect of DMF treatment on activated cells, HBMVEC were pretreated with DMF (1 h) prior to TNF treatment and compared to TNF treatment alone, which resulted in dysregulation of 348 genes (fold change > 2, FDR ≤ 0.01, p < 0.005, Table 1, see Additional file 3 for complete gene list).

**Table 1:** Differentially expressed HBMVEC transcripts in DMF pretreated TNF activated compared to TNF activated cells

Gene symbol	Description	Fold change	FDR p-value
STC1	Stanniocalcin 1	58	2.27E−07
SRXN1	Sulfiredoxin 1	8	6.99E−06
RGCC	Regulator of cell cycle	7	7.28E−07
HMOX1	Heme oxygenase 1	6	1.66E−05
NID2	Nidogen 2 (osteonidogen)	5	4.89E−06
OSGIN1	Oxidative stress induced growth inhibitor 1	5	2.88E−06
SLC16A6	Solute carrier family 16, member 6	5	7.07E−06
AHR	Aryl hydrocarbon receptor	4	9.25E−06
CCRL2	Chemokine (C–C motif) receptor-like 2	3	7.18E−05
CLDN14	Claudin 14	3	0.0022
VCAM1	Vascular cell adhesion molecule 1	− 488	9.19E−10
TNFAIP6	Tumor necrosis factor, alpha-induced protein 6	− 42	7.56E−08
SELE	Selectin E	− 20	9.64E−07
IL18R1	Interleukin 18 receptor 1	− 9	4.46E−06
OAS1	2–5-Oligoadenylate synthetase 1	− 9	2.53E−06
ICAM1	Intercellular adhesion molecule 1	− 6	5.61E−06
LTB	Lymphotoxin beta (TNF superfamily)	− 4	7.93E−06
NALCN	Sodium leak channel, non selective	− 4	0.0001
IL-6	Interleukin 6	− 3	0.0002
IL1R1	Interleukin 1 receptor, type I	− 3	0.0024

Selected transcripts shown. Fold change was calculated as the average between groups of log2-transformed data

**Fig. 2:**
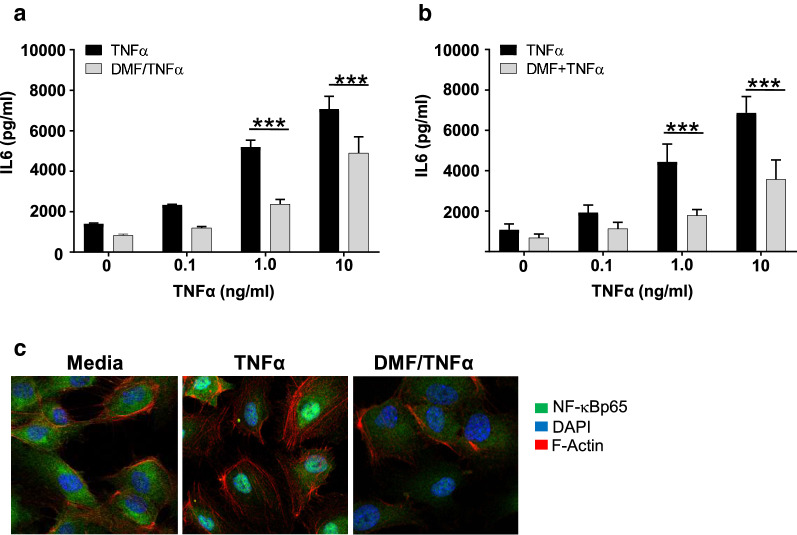
DMF treatment reduces activation of TNF stimulated HBMVEC. **a** IL-6 concentrations were measured by ELISA in the supernatants of HBMVEC stimulated by TNF for 24 h with or without 1 h DMF-pretreatment (n = 3 independent experiments in triplicate, ***p < 0.001, analysis of variance followed by post-hoc multiple comparisons using Turkey’s test). **b** IL-6 concentration measured by ELISA in the supernatants of HBMVEC stimulated by co-treatment with TNF and DMF for 24 h (n = 3 independent experiments in triplicate, test statistics as above). Bars in **a** and **b** depict the mean with SEM. **c** Confocal microscopy of NFκBp65 in resting or TNF stimulated (30 min) with or without DMF pre-treatment (1 h). Images are representative of 3 independent experiments. *DMF* dimethyl fumarate

DMF pretreatment prior to TNF stimulation increased transcript levels of several genes found in antioxidant pathways, including heme oxygenase 1 (HMOX1), sulfiredoxin 1 (SRXN1) and oxidative stress induced growth inhibitor 1 (OSGIN1) (Table 1, fold change > 2, FDR ≤ 0.01, p < 0.005, see Additional file 3 for complete list of pathways) as well as the NRF2-mediated oxidase stress response (Fig. 1b, p < 0.01, Z score at least 2 SD above the mean, Additional file 4: Table S4). Extracellular signal-regulated kinase 5 (ERK5) pathway, which is induced in response to oxidative stress is a central mediator of cell survival and apoptotic regulation, was also upregulated [53]. Moreover, DMF pretreatment significantly downregulated VCAM-1 and the parasite cytoadhesion receptor ICAM1, as well as transcripts involved in neuroinflammation signaling (IL-6, TREM1, PPAR, NFκB) (Fig. 1b, p < 0.01, Z score at least 2 SD above the mean, see Additional file 4 for complete gene list). IL-6, an inflammatory cytokine, and a marker of cellular activation levels in the HBMVEC supernatant to confirm the transcriptional data was measured. IL-6 levels increased in a TNF dose dependent manner. IL-6 concentration was significantly less in the supernatant of the DMF pretreated, TNF activated cells (Fig. 2a) compared to TNF activated cells. Moreover, when DMF was added simultaneously with TNF stimulation of HBMVEC, IL-6 levels were significantly reduced (Fig. 2b). To corroborate the DMF inhibitory effect on the NFκB pathway in HBMVEC, NFκB cellular localization by confocal imaging was performed. TNF stimulation resulted in translocation of NFκB from the HBMVEC cytoplasm to the nucleus as expected. In contrast, 1 h pretreatment with DMF treatment inhibited the TNF induced nuclear translocation of NFκB (Fig. 2c).

### Effect of DMF on the surface expression of parasite cytoadhesion receptors in HBMVEC

**Fig. 3:**
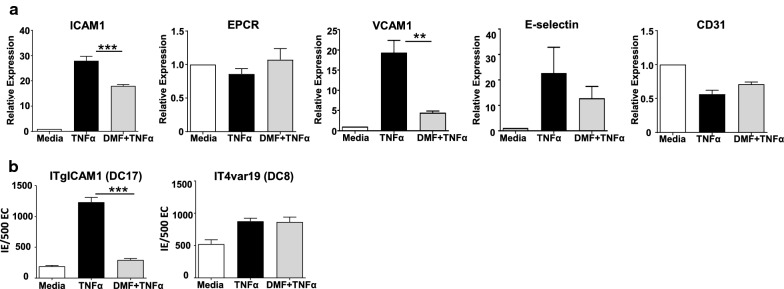
DMF down regulates ICAM1 expression and ITgICAM1 binding to HBMVEC. **a** Surface expression of ICAM1, EPCR, VCAM1, E selectin and CD31 were measured by flow cytometry on resting or TNF treated HBMVEC for 8 h with or without 6 h-pretreatment with DMF (n = 3–5 independent experiments). Fold expression relative to unstimulated cells are shown. Results were analysed using analysis of variance followed by post-hoc multiple comparisons using Sidak’s test. **p < 0.001, ***p < 0.0001. **b** Cytoadherence of laboratory strains ITgICAM1 (DC17) and IT4var19 (DC8) to primary HBMVEC was measured by semi-static binding assay, numbers of infected erythrocytes (IE) bound to 500 endothelial cells (EC) are shown. HBMVEC were left untreated, or stimulated with TNF (10 ng/ml), or DMF (50 μM) added 6 h prior to TNF for 20 h prior to parasite binding (n = 2–5 independent experiments, **p < 0.01, ***p < 0.001, analysis of variance followed by post-hoc multiple comparisons using Turkey’s test). Bars in **a** and **b** depict the mean with SEM

Previous work from a paediatric CM population has shown that circulating *P. falciparum*-IE utilize ICAM1 and EPCR to bind to primary HBMVEC [24]. During malaria infection, there is widespread upregulation of vascular endothelial ICAM1 due to cytokines such as TNF [54]. To study whether DMF can modulate the expression levels of parasite cytoadhesion receptors, HBMVEC were treated with TNF plus or minus pre-treatment with DMF. Whereas TNF increased the HBMVEC expression of ICAM1, VCAM1, E-selectin and decreased expression of EPCR surface levels quantified by flow cytometry, there were no changes in the constitutive endothelial marker CD31. DMF pretreatment strongly attenuated the TNF-induced upregulation of ICAM1, and VCAM1 (p < 0.01, one-way ANOVA test) (Fig. 3a), it did not significantly alter EPCR, E-selectin or CD31 surface expression levels.

### *Plasmodium falciparum* binding to HBMVEC after TNF activation and DMF treatment

To determine whether DMF could modify IE binding to TNF-stimulated HBMVEC, the binding of two long-term cultivated parasite lines with known adhesion traits for ICAM1 and EPCR was examined. Confluent monolayers of HBMVEC were stimulated with TNF for 20 h. As expected, there was a major binding increase (~ fivefold) in the ITgICAM1 parasite line (ICAM1 binder) to TNF-activated HBMVEC (Fig. 3b). By comparison, there was a non-significant increase (~ 1.5-fold) in IT4var19 (EPCR binder) parasite line (Fig. 3b). The respective ICAM1 and EPCR-binding dependencies of the ITgICAM1 and IT4var19 parasite lines to HBMVEC was confirmed using blocking antibodies (see Additional file 5). DMF pretreatment abolished the increased binding of the ITgICAM1 parasites (p < 0.0001, one-way ANOVA, Fig. 3b) and had no effect on the binding of IT4var19 parasites to TNF activated HBMVEC (p = 0.99, one-way ANOVA).

### Binding of CM derived *P. falciparum* isolates to HBMVECs

**Fig. 4:**
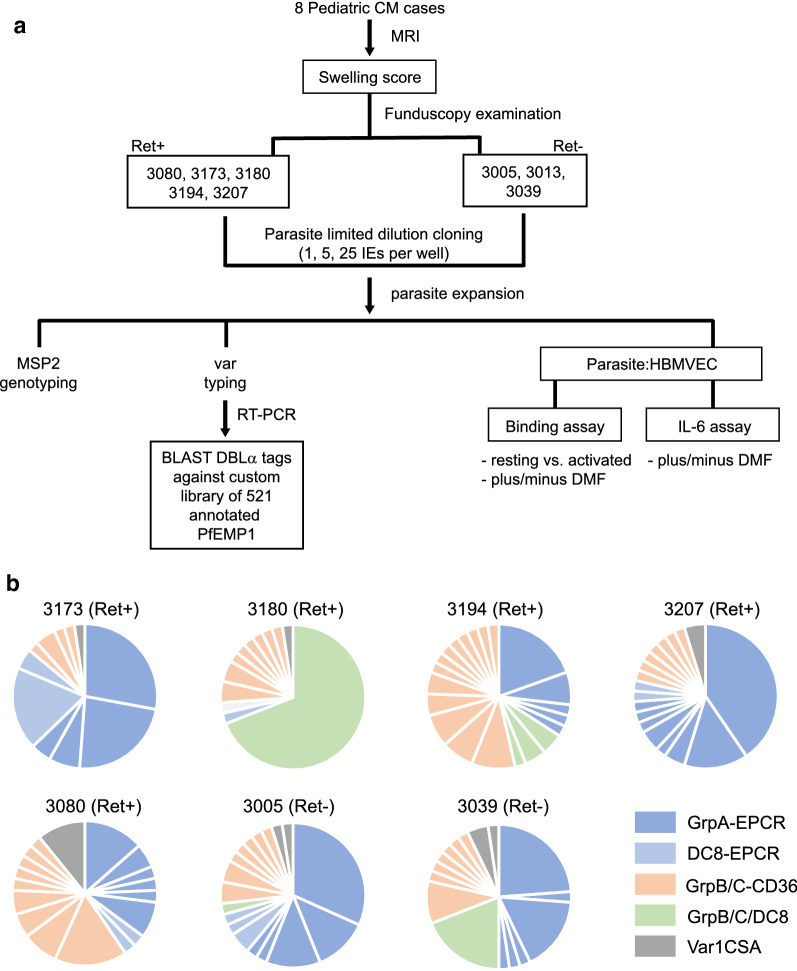
Erivation of CM parasite lines. **a** Parasite lines were derived by limited dilution cloning from 5 Ret+ cases and 3 Ret− cases. After approximately 30 or more cycles of growth and expansion, the parasite lines underwent MSP2 genotyping and *var* transcriptional profiling at the same time as the parasite-HBMVEC co-culture experiments. **b** Frequencies of distinct *var* DBLα tags expressed in the CM derived parasite lines at the time of co-culture experiments. The DBLα tags were subclassified based on BLAST identity to previously annotated full-length *var* genes and by the number of cysteine residues in the DBLα tag region (see Additional file 6)

The binding of parasite lines derived from Malawian children with CM to HBMVEC (Fig. 4a) was then examined. Parasite lines were derived from 8 comatose children in the research ward of the BMP who were enrolled in 2011–2014. Bacteraemia was excluded in all by blood culture evaluation of the admission blood sample. Five of the parasite lines were from malaria retinopathy positive (Ret+) children (3173, 3180, 3194, 3207 and 3080) and three were from Ret− children (3005, 3013, and 3039). All patients had Blantyre Coma Score of ≤ 2 and anaemia upon admission to the BMP and sustained prolonged coma (Table 2). All patients survived.

**Table 2:** Description of children with CM where *P. falciparum* lines were derived

Parasite line	Retinopathy status	Blantyre coma score	Coma duration (h)	Brain swelling score (MRI^a^)	Parasitemia (IE/µl)	HCT (% volume)
3173	Ret+	2	36	5	148,500	19
3180	Ret+	2	16	3	296,000	32
3194	Ret+	2	20	ND	177,120	23
3207	Ret+	1	108	3	632,500	21
3080	Ret+	2	56	5	520	21
3005	Ret−	2	24	ND	20,500	25
3013	Ret−	1	10	3	45,000	25
3039	Ret−	2	12	ND	600,000	25

*Ret* retinopathy, *HCT* hematocrit, *ND* not done

^a^MRI scores for brain swelling (scale 1–8): 1–4: absence of swelling, 5–6: mild/moderate swelling; 7–8: severe swelling

By MRI, two patients had mild to moderate severe brain swelling. After limited dilution cloning and in vitro expansion, 7 of 8 clonal parasite lines had a single MSP2 sequence and one parasite line (3005) had two MSP2 genotypes, indicating it was multi-clonal.

Previously, it has been shown that EPCR-binding *var* transcripts (DC8 *var* and group A *var*) are increased in IE from paediatric CM patient cohorts [23]. RNA was isolated from the laboratory adapted CM parasite isolates and sequenced DBLα tags. All eight CM parasite lines expressed a mixture of *var* transcripts, including predicted EPCR binding (DC8 and group A) and CD36 binding transcripts (group B and C) (Fig. 4, see Additional file 6). The number of different *var* transcripts ranged from 7 to 21 per CM parasite line, indicating that the parasite lines had likely undergone *var* gene switching during cultivation following the limited dilution cloning. As observed previously in this patient population [23] there was a high degree of genetic diversity between DBLα tags amplified from the 8 CM parasite lines. Indeed, only one tag was identical between two parasite lines (3194D and 3207s15, predicted CD36 binder).

**Fig. 5:**
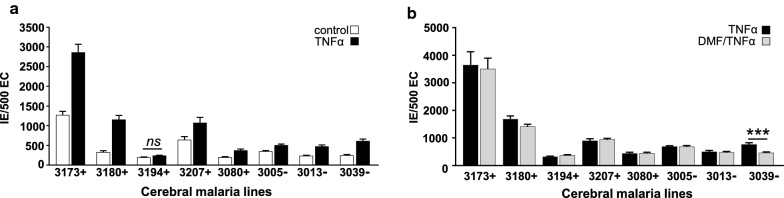
Increased CM parasite cytoadherence to TNF-stimulated HBMVEC, without change in binding in DMF pre-treated cells. **a** Cytoadherence of 8 CM parasite lines to resting HBMVEC or after 24 h of stimulation with TNF (10 ng/ml). Numbers of *P. falciparum*-IE bound to 500 endothelial cells (EC) are shown. All isolates had increased binding to TNF stimulated HBMVEC except where noted (n = 2–3, p < 0.0001, Wilcoxon matched-pairs signed rank test). **b** Cytoadherence of 8 CM parasite lines to resting and TNF stimulated primary HBMVEC with or without 6 h of DMF pre-treatment, numbers of IE bound to 500 EC are shown (n = 2–5 independent experiments, ***p < 0.001, analysis of variance followed by post-hoc multiple comparisons using Turkey’s test). Bars in **a** and **b** depict the mean with SEM variance

The binding of CM derived isolates to HBMVEC was measured. At the time of the binding assay, all the parasite lines expressed a high proportion of group A or DC8-EPCR binding *var* transcripts (range 33–88%, Fig. 4b). Additionally, group A or DC8-EPCR binding *var* transcripts were the dominant transcripts (> 50% transcripts) in all the parasite lines, except for 3194 (Fig. 4b). The binding levels of the eight CM-derived parasite lines ranged from 191 to 1270 IE per 500 resting HBMVEC (Fig. 5a). TNF activation of HBMVEC induced a significant increase in cytoadherence of 7/8 CM parasite lines (p = 0.0020, Wilcoxon matched-pairs signed rank test) (Fig. 5a, range 237 to 2860 IEs per 500 HBMVEC). There was no significant differences between isolate binding based on donor retinopathy status (baseline binding, p = 0.60 Mann–Whitney test) to either resting or TNF stimulated HBMVEC (p = 0.60 Mann–Whitney test). DMF pre-exposure was tested to determine if it reduced the binding of CM derived isolates to TNF-stimulated HBMVEC. Parasite line 3039 demonstrated reduced binding under DMF treatment (p < 0.001) however, overall DMF did not reduce binding levels of parasite lines to activated HBMVEC (Fig. 5b, p = 0.99, one-way ANOVA).

### DMF modulates HBMVEC activation induced by TNF and parasite exposure

**Fig. 6:**
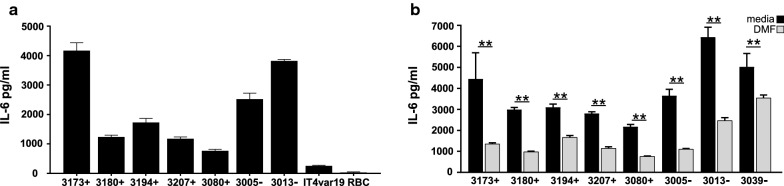
Differential activation of HBMVEC by co-culture with CM parasite lines is reduced by DMF pretreatment. **a** IL-6 concentrations were measured by ELISA in the supernatants of HBMVEC stimulated by direct contact with *P. falciparum*-IE from 8 CM parasite lines, IT4var19, and uninfected red blood cells (RBC) at IE:HBMVEC ratio of 12:1 for 24 h. **b** IL-6 levels of HBMVEC supernatants co-cultured with IE for 24 h with or without 1 h DMF pretreatment (n = 2 independent experiments in triplicate). Results were analysed using analysis of variance followed by post-hoc multiple comparisons using Turkey’s test. **p < 0.01. Bars depict the mean with SEM

Previous work has shown that IEs can activate endothelial cells to secrete proinflammatory cytokines, such as IL-6 [28, 52]. To test the effect of DMF to modulate endothelial cell activation by parasites, IE were co-incubated with HBMVEC for 24 h, which allowed the CM parasite lines to cycle through schizogony with associated erythrocyte haemolysis. Consistent with previous findings the IT4var19 parasite line induced minimal IL-6 release from primary HBMVEC (Fig. 6a) [55]. By comparison, the CM parasite lines induced a range of IL-6 release (from 758 to 4158 pg/ml) (Fig. 6a). DMF inhibited the release of IL-6 in all the HBMVEC parasite co-cultures, on average by two-fold with mean IL-6 levels decreased from 3824 to 1629 pg/ml (p < 0.001) (Fig. 6b).

## Discussion

Brain microvasculature pathology is central in CM and contributes to CNS damage and death. Systemic toxicity persists after artemisinin administration with protracted fever and coma duration and in some cases worsening brain edema that can lag after administration of anti-malarial therapy [13]. Reduction of CNS endothelial cell toxicity induced by host and parasite molecules could improve outcomes. DMF reduces TNF or CM parasite line activation of primary brain HBMVEC via multiple protective pathways and potentially provides a new therapeutic approach to improve CM outcomes.

The protective effects of the Nrf2 pathway has been harnessed clinically with DMF to treat multiple sclerosis and severe psoriasis [56, 57]. In models of brain ischaemia, DMF attenuates brain edema, stabilizes the BBB by preventing disruption of endothelial tight junctions and reduces neurological deficits via anti-inflammatory, anti-oxidant, and cellular restorative pathways present in human endovascular and immune systems [35, 36, 58, 59]. Cerebral vascular disease and CM have shared pathophysiology which motivated this study of the potentially protective effects of DMF on HBMVEC which have a specialized role to form an interface between blood and neural tissue through the BBB [60]. To mimic in vivo conditions of CM, HBMVEC were activated with TNF treatment [61, 62]. TNF predictably upregulated many inflammatory pathways and drove NFκB nuclear translocation in HBMVEC, consistent with previous studies [63].

DMF countered these pro-inflammatory processes and there is evidence in murine experimental cerebral malaria (ECM) model that some of these DMF modified genes may be protective. DMF reduced inflammasome signaling, with down regulation of NLR Family Pyrin Domain Containing 3 (NLRP3) and IL1β. Reduction of inflammasome activation and IL1β production in microglia and intracerebral monocytes enhanced recovery in the ECM model [64]. Whether DMF induces this protective program in neural cells and monocytes would need further investigation. HMOX1 upregulated by DMF, can degrade heme and confer neuroprotective effects, and HMOX1 upregulation in the ECM model prevented BBB disruption and increased survival [65, 66]. DMF treatment of HBMVEC upregulated the PPAR pathway, this may provide the protection seen where a PPAR agonist protected against endothelial cell activation to enhance BBB integrity and improve neurocognitive outcomes and survival in ECM [67]. ErbB4 and Neuregulin Signaling were upregulated with DMF treatment. ErbB4 is an epidermal growth factor receptor kinase which been shown to preserve BBB integrity after subarachnoid hemorrhage in rats [68]. These pathways may be protective as an in increase in ErbB4 phosphorylation by Neuregulin-1β (NRG-1) treatment reduces mortality in the ECM model [69].

Other DMF regulated pathways may also have a role in neuroprotection. For example, the pro-inflammatory cytokine IL-6, which typically promotes vasoconstriction, ROS production, and BBB disruption is induced by both TNF and in parasite co-culture, and its induction was reduced in both circumstances with DMF treatment [70–72]. The extracellular signal-regulated kinase (ERK) 5, a member of the mitogen-activated protein (MAP) kinase super family was also upregulated under DMF treatment. ERK5 stimulates tight junction formation and reduces permeability in cardiac endothelial cells and potentially serves a similar function in brain endothelial cells [73, 74]. The inducible nitric oxide synthase (iNOS) generates nitric oxide (NO), which is neurotoxic and may be relevant to CM as it has been found to be increased in neurons, astrocytes and microglial cells in patients with CM compared to non-CM control brains [18, 75]. DMF inhibits iNOS expression in LPS activated microglia and astrocytes in vitro model of brain inflammation [76]. Thus, numerous signaling pathways were initiated in DMF-treated cells that could potentially be beneficial in the context of CM.

To define the effect of DMF on parasite adherence to and activation of HBMVEC, an in vitro co-culture model was used. Previous work has established that ICAM1 and EPCR are important receptors for parasite binding to primary HBMVEC [22, 24, 77, 78]. The pro-inflammatory cytokine TNF increased the expression level of ICAM1 on endothelial cells as expected [79]. DMF treatment reduced ICAM1 upregulation on TNF-stimulated HBMVEC and inhibited the binding increase of a well-characterized ICAM-1 binding parasite line. In contrast there was a modest decrease in EPCR expression with TNF, consistent with prior studies [55] and no change in binding of an EPCR-binding parasite line with DMF treatment.

Under DMF pretreatment, the CM isolates behaved differently than the control parasite line that had dual binding activity for CD36 + ICAM-1. This difference may reflect the diverse multi-binding properties of the parasite lines. As DMF blunted ICAM1 upregulation, this finding suggests that DMF is less effective against parasite lines with dual EPCR + ICAM-1 binding or that some parasites may bind EPCR plus an unknown receptor, which is not modified by the DMF pretreatment. Thus, more work is needed to understand the co-receptor binding dependencies of parasite adhesion to brain endothelial cells.

During IE rupture, inflammatory products are released that can activate endothelial cells [28, 80]. Prior studies have shown that HBMVEC co-cultured with the *P. falciparum* 3D7 lab strain upregulates the NFκB pathway, increases IL-6 and IL1β levels and increases the expression of ICAM1, compared to controls [52]. CM derived parasite lines induced much higher levels of IL-6 secretion from HBMVEC than the long-term laboratory strain IT4var19. Similar to previous findings, the induction of IL-6 release varied between CM parasite lines and this variation in cellular activation may be due to parasite pathogenicity factors that mediate endothelial cell apoptosis [81]. Notably, DMF treatment significantly attenuated IL-6 release during parasite-HBMVEC co-culture and inhibited TNF upregulation of cell adhesion molecules.

One limitation of this study is that DMF treatment was added to HBMVEC before or simultaneously with TNF administration. To adequately recapitulate the clinical scenario where patients with CM present with an inflammatory state, studies of DMF in the ECM model will require that DMF administration is delivered after infection is established.

## Conclusion

This data suggests that DMF can harnesses multiple protective mechanisms to reduce HBMVEC activation due to TNF and IE to potentially reduce neurovascular damage during CM. This study also provides additional data of endothelial cell binding and activation of a unique panel of CM derived isolates with highly characterized binding domains. Studies of DMF administration after initiation of *Plasmodium berghei* (strain ANKA) infection in the ECM model are underway to determine if it can provide a survival advantage and prompt clinical trials to test DMF as an adjunctive therapy in CM to improve outcomes.

## Supplementary information

**Additional file 1.** Differentially expressed transcripts in TNF treated HBMVEC compared to media treatment. HBMVEC were incubated with TNF (10 ng/ml) or media alone for 6 h. Total RNA was extracted, reverse-transcribed, amplified and hybridized to a microarray. Gene expression profiles were generated using a robust multi-array average algorithm followed by quantile normalization and batch correction. Data represents three independent experiments, each done in triplicate. 514 transcripts were significantly differentially abundant between experimental groups using a Student’s t test and FDR (fold change > 2, FDR ≤ 0.01, p < 0.005).

**Additional file 2.** Differentially expressed pathways in TNF treated HBMVEC compared to media treatment. HBMVEC incubated with TNF (10 ng/ml) or media alone for 6 h. Total RNA was extracted, reverse-transcribed, amplified and hybridized to a microarray. Gene expression profiles were generated using a robust multi-array average algorithm followed by quantile normalization and batch correction. Data represents three independent experiments, each done in triplicate. 41 Ingenuity Canonical Pathways were significantly differentially expressed with p value calculated by a right tailed Fisher’s exact test. (p < 0.01, Z score at least 2 SD above the mean).

**Additional file 3.** Differentially expressed transcripts in DMF pretreated TNF treated HBMVEC compared to TNF treatment. HBMVEC were preincubated with DMF (50 μM) for 1 h prior to the addition of TNF incubation (10 ng/ml) or TNF incubation alone for 6 h. Total RNA was extracted, reverse-transcribed, amplified and hybridized to a microarray. Gene expression profiles were generated using a robust multi-array average algorithm followed by quantile normalization and batch correction. Data represents three independent experiments, each done in triplicate. 348 transcripts were significantly differentially abundant between experimental groups using a Student’s t test and FDR (fold change > 2, FDR ≤ 0.01, p < 0.005).

**Additional file 4.** Differentially expressed pathways in DMF pretreated TNF treated HBMVEC compared to TNF treatment. HBMVEC were preincubated with DMF (50 μM) for 1 h prior to the addition of TNF incubation (10 ng/ml) or TNF incubation alone for 6 h. Total RNA was extracted, reverse-transcribed, amplified and hybridized to a microarray. Gene expression profiles were generated using a robust multi-array average algorithm followed by quantile normalization and batch correction. Data represents three independent experiments, each done in triplicate. 76 Ingenuity Canonical Pathways were significantly differentially expressed with p value calculated by a right tailed Fisher’s exact test. (p < 0.01, Z score at least 2 SD above the mean).

**Additional file 5.** Binding specificity of *P. falciparum *parasites ITgICAM1 and IT4var19 to HBMVEC. A. Binding of ITgICAM1 (DC17) IE to resting and TNF activated HBMVEC is shown as percent binding to HBMVEC relative to unstimulated HBMVEC. Inhibitory antibodies against ICAM1 (mAb15.2) or IgG isotype control were added to HBMVEC monolayers prior to IE cytoadherence. B. Binding of IT4var19 (DC8) IE to resting HBMVEC is shown as percent binding to HBMVEC. Inhibitory antibodies against EPCR (mAb252) or IgG isotype control were added to HBMVEC monolayers prior to IE cytoadherence (n = 3 independent experiments). Results shown were analysed using analysis of variance followed by post-hoc multiple comparisons using Turkey’s test, ***p < 0.001

**Additional file 6.** List of DBLa tags from CM parasite lines at time of binding assay. DBLα tags from each parasite line one day prior to the HBMVEC cytoadherence experiments are reported. Total ring stage RNA was extracted, reverse transcribed to cDNA and 40-50 DBLα amplicons were PCR-amplified and sequenced from each CM parasite line. BLAST searches against a custom library of 521 annotated *var* genes classified DBLα tags into predicted CD36 or EPCR binders. Predictions were considered high confidence when ≥ 4 of the top 5 hits were the same type. The number of cysteine residues in the DBLα amplicons were quantified.

## Data Availability

Parasite isolate *var* transcript sequences will be available upon request.

## Abbreviations

BBB: Blood brain barrier
CM: Cerebral malaria
DMF: Dimethyl fumarate
ECM: Experimental cerebral malaria
EPCR: Endothelial protein C receptor
HBMVEC: Human brain microvascular endothelial cell
ICAM1: Intercellular adhesion molecule 1
IE: Infected erythrocyte
PCA: Principal component analysis
RBC: Red blood cell
TNF: Tumour necrosis factor
