# Supplementary figures and images for: Dimethyl fumarate reduces TNF and Plasmodium falciparum induced brain endothelium activation in vitro
Source: Malar J. 2020 Oct 21;19:376. doi: 10.1186/s12936-020-03447-7 (PMC7579885; doi:10.1186/s12936-020-03447-7)

A

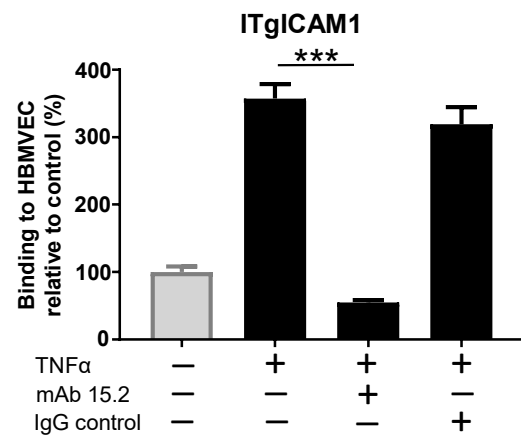

B

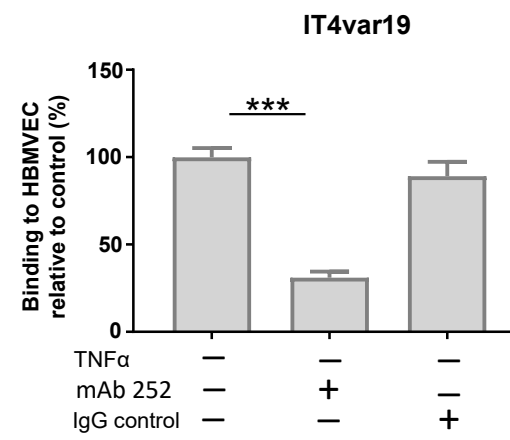

Supplement: Supplementary file 5 — Additional file 5. Binding specificity of P. falciparum parasites ITgICAM1 and IT4var19 to HBMVEC. A. Binding of ITgICAM1 (DC17) IE to resting and TNF activated HBMVEC is shown as percent binding to HBMVEC relative to unstimulated HBMVEC. Inhibitory antibodies against ICAM1 (mAb15.2) or IgG isotype control were added to HBMVEC monolayers prior to IE cytoadherence. B. Binding of IT4var19 (DC8) IE to resting HBMVEC is shown as percent binding to HBMVEC. Inhibitory antibodies against EPCR (mAb252) or IgG isotype control were added to HBMVEC monolayers prior to IE cytoadherence (n = 3 independent experiments). Results shown were analysed using analysis of variance followed by post-hoc multiple comparisons using Turkey’s test, ***p < 0.001 [file 12936_2020_3447_MOESM5_ESM.pdf]
